# Realizing Full Coverage of Stable Perovskite Film by Modified Anti-Solvent Process

**DOI:** 10.1186/s11671-017-2117-6

**Published:** 2017-05-22

**Authors:** Long Ji, Ting Zhang, Yafei Wang, Peng Zhang, Detao Liu, Zhi Chen, Shibin Li

**Affiliations:** 1 State Key Laboratory of Electronic Thin Films and Integrated Devices, and School of Optoelectronic Information, University of Electronic Science and Technology of China (UESTC), Chengdu, Sichuan 610054 China; 2 Department of Electrical and Computer Engineering and Center for Nanoscale Science and Engineering, University of Kentucky, Lexington, Kentucky 40506 USA

**Keywords:** Lead-free perovskite solar cells, Solvent engineering, Anti-solvent dripping

## Abstract

Lead-free solution-processed solid-state photovoltaic devices based on formamidinium tin triiodide (FASnI_3_) and cesium tin triiodide (CsSnI_3_) perovskite semiconductor as the light harvester are reported. In this letter, we used solvent engineering and anti-solvent dripping method to fabricate perovskite films. SnCl_2_ was used as an inhibitor of Sn^4+^ in FASnI_3_ precursor solution. We obtained the best films under the function of toluene or chlorobenzene in anti-solvent dripping method and monitored the oxidation of FASnI_3_ films in air. We chose SnF_2_ as an additive of CsSnI_3_ precursor solution to prevent the oxidation of the Sn^2+^, improving the stability of CsSnI_3_. The experimental results we obtained can pave the way for lead-free tin-based perovskite solar cells (PSCs).

## Background

Organic-inorganic halide perovskite solar cells have attracted great attention in recent years. The general formula for perovskite is ABX_3_ (A cation, B cation, X anion). In 2012, the first all-solid-state solar cell [1] was introduced with a power conversion efficiency (PCE) of 9% [1] which is now increasing up to 22% [2]. These perovskite solar cells are mainly based on methylammonium lead iodide (MAPbI_3_) [3–8] and formamidinium lead iodide (FAPbI_3_) [9, 10]. Different halogens are used as anions (I, Br, Cl) [11] and inorganic cesium (Cs) is also used as cation with methylammonium(MA) and formamidinium(FA) in perovskite solar cells (PVCs) [12]. All of these materials have toxic lead, which is harmful to human health. This limits the commercial use of perovskite solar cells. Scientists have been looking for a non-toxic elements to replace lead in perovskites [16–26]. Some tried to mix the bivalent cations Sn^2+^ and Pb^2+^ as CH_3_NH_3_Sn_x_Pb_(1−x)_I_3_ [13, 14] and others mixed the monovalent A cations along with mixed bivalent cations, i.e. FA_0.8_MA_0.2_Sn_x_Pb_1−x_I_3_ [15], but these perovskites are still toxic. In 2014 [16] Snaith and co-workers first developed complete lead-free perovskite solar cells based on methylammonium tin triiodide (CH_3_NH_3_SnI_3_) and achieved a PCE around 6%. In the same year, Kanatzidis and co-workers [17] investigated CH_3_NH_3_SnI_3-x_Br_x_ and received almost the same PCE. MASnI_3_ is very unstable in air according to previous reports [16]. Later, researchers attempted to use formamidinium tin triiodide (FASnI_3_) with the additive of SnF_2_ to delay the oxidation of Sn^2+^ to Sn^4+^ [18, 19]. They found that FASnI_3_ was more stable than MASnI_3_. Recently, Seok and co-workers [20] obtained a smooth and dense FASnI_3_ perovskite layer using SnF_2_-pyrazine complex as an additive. With SnF_2_ additives and diethyl ether dripping in a solvent engineering process to synthesize FASnI_3_ perovskite thin films [21], Dewei Zhao and co-workers [21] achieved an improved PCE up to 6.22% for lead-free Sn-based perovskite solar cells .

Similar as FASnI_3_, CsSnI_3_ also shows perovskite phase at room temperature. CsSnI_3_ has four phases at different temperatures [22], but only the black orthorhombic phase B-γ-CsSnI_3_ is the perovskite phase. Kumar et al. [23] fabricated solar cells using CsSnI_3_ as the perovskite layer between a TiO_2_ electron transfer layer and a Spiro-OMeTAD hole transfer layer and achieved a PCE of 2%. Zhou et al. [24] modulated B-γ-CsSnI_3_ grain sizes by employing different annealing temperatures and chose the optimal architecture for perovskite solar cells. They achieved a PCE of 3.31%. Marshall et al. [25] proved that the existence of SnCl_2_ resulted in higher film stability and they achieved a PCE of 3.56% from PSCs without any hole-selective interfacial layer. CsSnI_3_ can be used as the perovskite absorption layer as well as the active region in lead-free perovskite infrared LEDs [26]. Generally, Spiro-OMeTAD is used as a hole transport material (HTM), which typically contains acetonitrile and lithium (Li) and/or cobalt (Co) salts that may change the morphology of Sn-based perovskite films and form the undesirable Cs_2_SnI_6_ polymorph [21, 22].

While SnF_2_-pyrazine complex [20] and SnF_2_ [21] have been used as additives into FASnI_3_ solution and resulted in good performance on stability and efficiency, SnCl_2_ also can be used as an alternative additive. The mechanism is similar to other tin halides (SnF_2_, SnCl_2_, SnBr_2_, SnI_2_) additives which are chosen in CsSnI_3_-based perovskite photovoltaics [25]. In this report, we chose SnCl_2_ as an additive of FASnI_3_ solution and SnF_2_ as an additive of CsSnI_3_ solution to investigate the stability of the perovskite films, respectively. The measurement of the evolution of absorption spectra at different time courses and other experimental results (SEM, photos etc.) showed that the stability of the films was improved by both additives. With different anti-solvent dripping during spin-coating, we obtained some new findings about the surface morphology and obtained complete coverage of perovskite films.

## Methods

The synthesis method of FASnI_3_ follows reference [21]: 372 mg of SnI_2_ (Sigma-Aldrich) and 172 mg of formamidinium iodide (FAI) were dissolved in 800 μl anhydrous dimethylformamide (DMF, Sigma-Aldrich) and 200 μl anhydrous dimethyl sulfoxide (DMSO, Sigma-Aldrich). For this precursor solution, 10 mol% SnCl_2_ was added and then stirred. Via spray pyrolysis at 500 °C, a compact layer of TiO_2_ substrate was deposited on FTO glass. The films were annealed at 500 °C for 15 min and then cooled down to room temperature. The mesoporous TiO_2_ scaffold was spin-coated at 4500 rpm for 20 s and then heated at 500 °C for 1 h. FASnI_3_ films were synthesized by spin-coating the precursor solution with SnCl_2_ additives at 4000 rpm for 60 s in a glove box. During the process of spin-coating, the anti-solvent (diethyl ether, toluene, chlorobenzene) was dripped and then the perovskite films were annealed at 70 °C for 20 min.

The synthesis method of CsSnI_3_ is described in a previous paper [23]: 0.6 M of CsSnI_3_ that contained equimolar quantities of CsI and SnI_2_ without or with 10 mol% SnF_2_ additives, respectively, was added to DMSO and stirred overnight at 70 °C. Sixty microliters of the precursor solution was spin coated onto the TiO_2_ substrate at 4000 rpm. The substrates were then annealed at 70 °C for 10 min, and mirror-like black perovskite films were formed.

## Results and Discussion

**Fig. 1:**
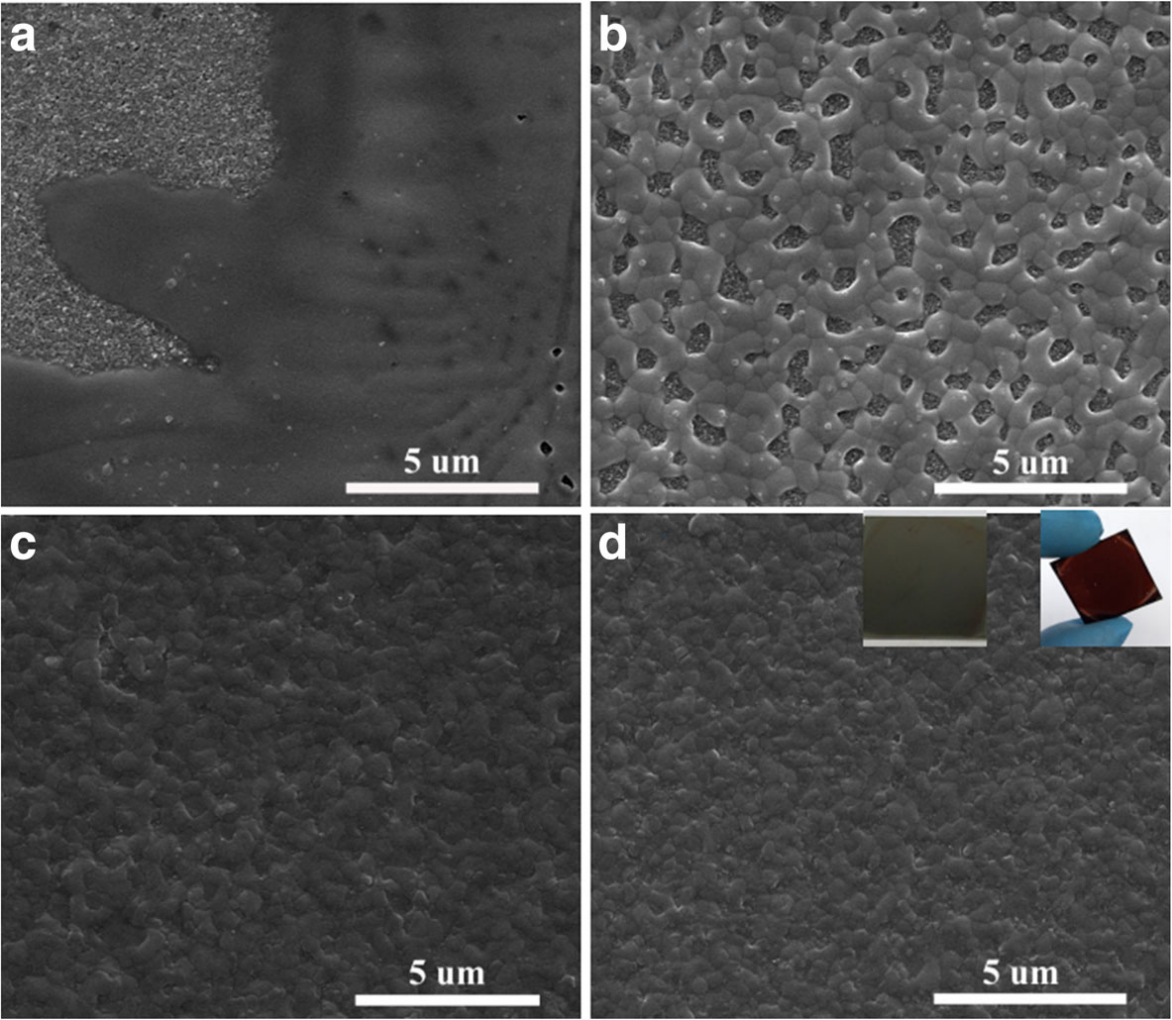
SEM images of perovskite films prepared (**a**) without anti-solvent dripping, (**b**) with diethyl ether dripping, (**c**) with toluene dripping, and (**d**) with chlorobenzene dripping

Under the effect of different anti-solvent dripping, the FASnI_3_ films with 10 mol% SnCl_2_ additives exhibit different film morphologies. Figure 1 shows the SEM images of FASnI_3_ perovskite films on TiO_2_ with different anti-solvents dripped. Figure 1a shows discontinuous nucleation, partial coverage and the presence of pinholes on the surface of the FASnI_3_ film without (w/o) any anti-solvent dripped. Dripping with diethyl ether (Fig. 1b), the formed FASnI_3_ film likes a net which has a lot of holes on it and spreads on the TiO_2_ substrate. Dripping with toluene or chlorobenzene (Fig. 1c and d, respectively), the surface morphology of the FASnI_3_ film has been further improved, and the film is highly uniform and dense with full coverage on the substrate. Using toluene as the anti-solvent, the average size of crystal particles is bigger than that of films fabricated by using chlorobenzene as the anti-solvent. These results are consistent with those reported in other articles [20, 21]. Without anti-solvent dripped, the film does not change color during spin-coating, and after continuous annealing at 70 °C, the film turns to black immediately and leads to the formation of a rough surface. When the film is dripped with diethyl ether, toluene or chlorobenzene, it changes into a reddish color immediately. After thermal annealing at 70 °C for 20 min, the film turns whitish with diethyl ether dripping and black (the left insert in Fig. 1d) with toluene or chlorobenzene dripping. No matter what kind of anti-solvent is dripped, films from the back view of the FTO glasses are brownish red (the right insert in Fig. 1d).

**Fig. 2:**
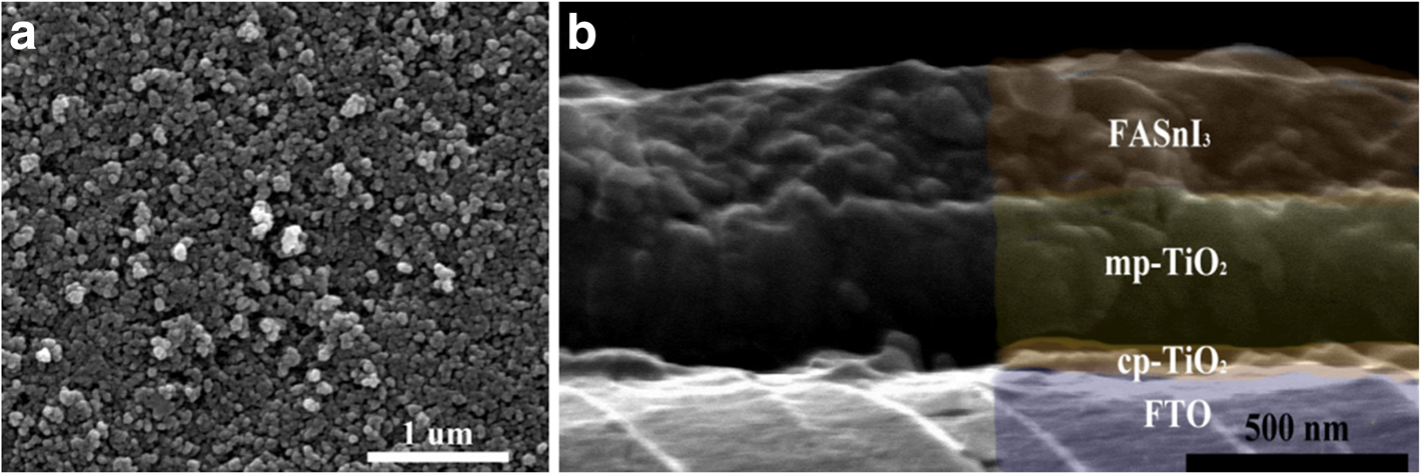
XRD patterns of FASnI_3_ perovskite films under different anti-solvents dripped

To investigate whether the anti-solvents dripping would lead to any crystal phase transition or not, we measured the XRD patterns. As shown in Fig. 2, all of the FASnI_3_ films that were formed on TiO_2_ crystallize in the orthorhombic structure and random orientation, which is consistent with other reports [20, 21].

**Fig. 3:**
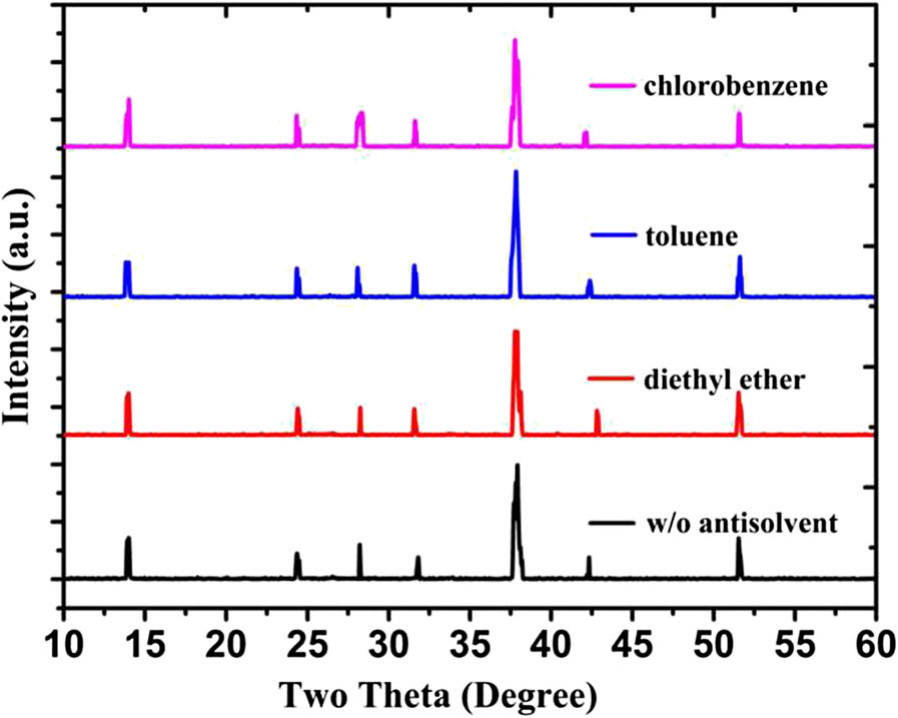
**a** SEM top view of TiO_2_ surface. **b** The cross-sectional SEM image of a structure of FTO/compact TiO_2_/mesoporous TiO_2_/FASnI_3_

Figure 3a shows the scanning electron microscopy (SEM) images of TiO_2_ substrates. The cross-sectional SEM image (Fig. 3b) of a structure of FTO/compact TiO_2_/mesoporous TiO_2_/FASnI_3_ clearly displays the stacked layers. From the figure, the thickness of FASnI_3_ is about 250 nm.

**Fig. 4:**
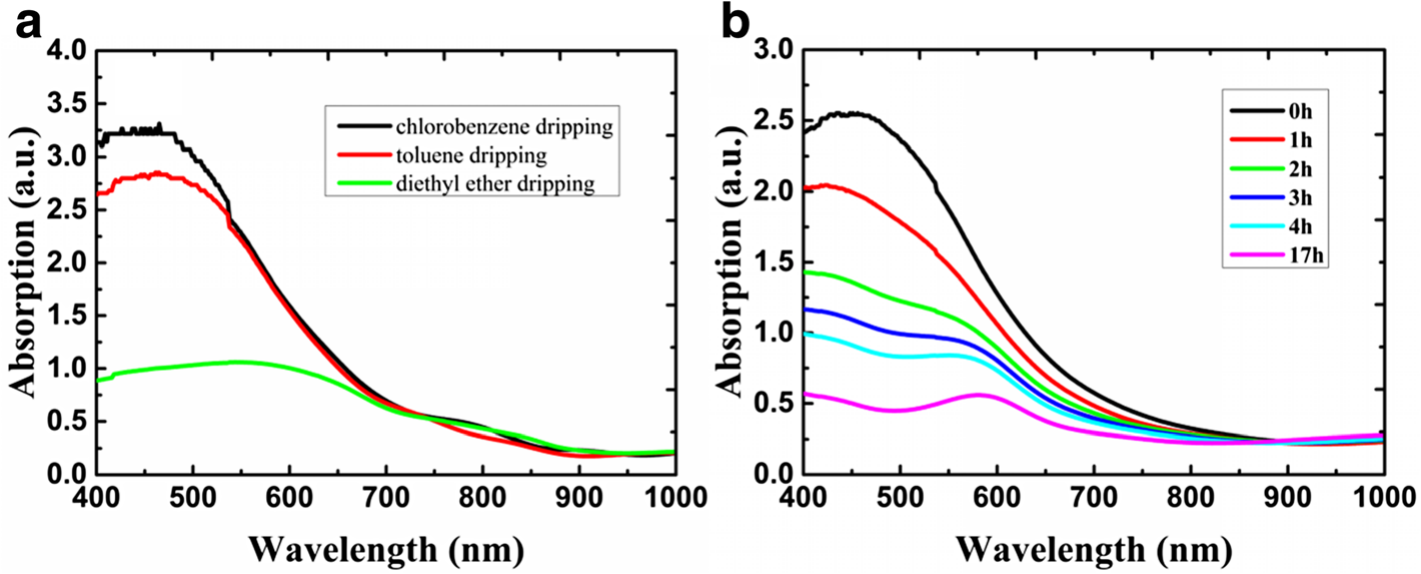
Absorption spectra of FASnI_3_ + 10% SnCl_2_ films (**a**) with different anti-solvents dripping on TiO_2_ and (**b**) with different time courses in air

The optical absorption spectra of the FASnI_3_ perovskite thin film with 10 mol% SnCl_2_ additives under the effect of different anti-solvent dripping are shown in Fig. 4a. The absorption onset occurs at 900 nm, and this result is consist with that reported by other groups [21]. As shown in Fig. 4, there are various absorption peaks of the films prepared by dripping with different anti-solvents. The absorption strength can indirectly reflect the quality of perovskite films. It is known that FASnI_3_ can automatically degrade to FA_2_SnI_6_ in air, [18, 21] and the absorption coefficient of the latter in the visible spectrum is smaller than that of the former. Degradation of the film in air with respect to time can be measured from absorption spectra. As shown in Fig. 4b, UV-vis absorption as a function of time was measured. The changed intensity of absorption reflected the process of degradation. Note that, relative humidity of the environment was about 46% and the room temperature was 15 °C. From optical pictures, we can see that FASnI_3_ degrades quickly during the first few hours. After 17 h, the absorption peak corresponding to FA_2_SnI_6_ becomes obvious. This result confirms that FASnI_3_ can degrade to FA_2_SnI_6_ in air.

**Fig. 5:**
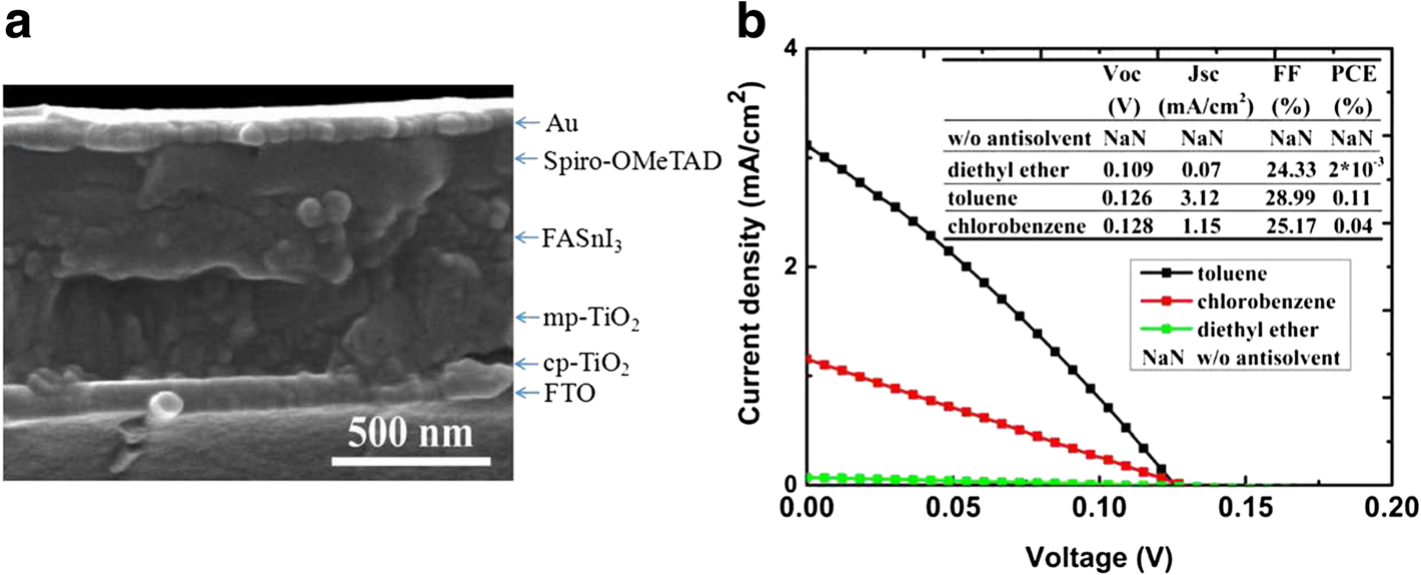
**a** Cross-sectional SEM image of a completed device. **b** J-V curves of the FASnI3 perovskite solar cells using different anti-solvents

Figure 5a shows a cross-sectional SEM image of the FASnI_3_ PSCs with a structure of FTO/cp-TiO_2_/mp-TiO_2_/FASnI_3_/Spiro-OMeTAD/Au. Figure 5b shows the J-V curves measured with different anti-solvent effects. Although the PCEs of these PSCs are quite low, some characteristics still offer new insights of the fabrication of lead-free perovskites. From Fig. 1a, we know that the nucleation of the film without anti-solvent dripping is discontinuous, which leads to nano-radiative recombination of electrons and holes and causes a large leakage current between TiO_2_ and FASnI_3_. The result of the films used diethyl ether as the anti-solvent was approximately the same as the untreated sample. The leakage current is large, but the coverage of the film is improved. With toluene and chlorobenzene as anti-solvents, the coverage of the film has been further improved, and larger crystal particles can create less grain boundaries to enhance the charge separation and collection of electrons and holes, thus leading to the highest PCE.

There are several challenges that hinder performance improvement of CsSnI_3_ perovskite solar cells: (1) Sn^2+^ oxidizes to Sn^4+^ easily, which seriously affects the photoelectric properties of CsSnI_3_ perovskite solar cells. (2) It is difficult to synthesize uniform and fully covered lead-free Sn-based thin films. Even with different additives, there are many pinholes existing on the crystallite surface which may short electron transfer layer and hole transport layer, leading to an enormous leakage current. (3) Lead-free Sn-based PSCs are often prepared in regular cell structures [18, 23]. In the following content, we have made some preliminary studies on the CsSnI_3_ films.

**Fig. 6:**
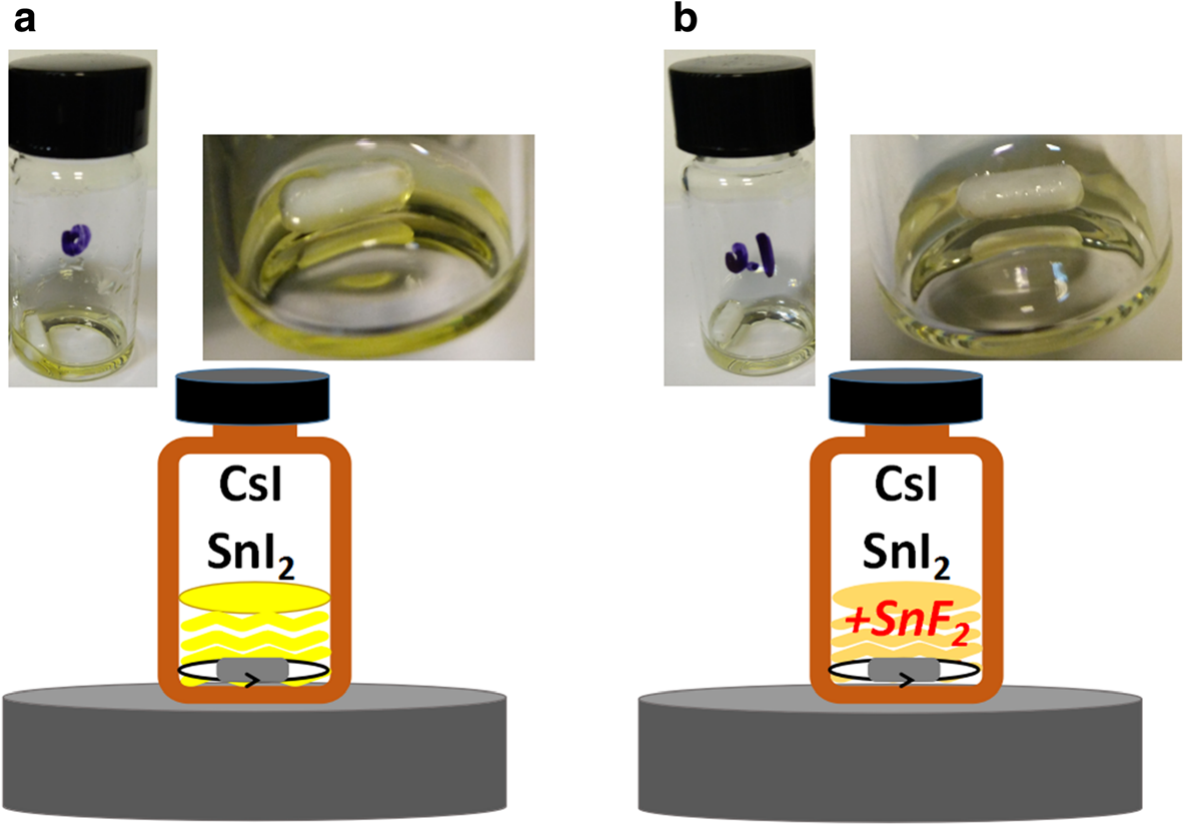
**a** Pure CsSnI_3_ without any additives. **b** 10 mol% SnF_2_ additives in CsSnI_3_ precursor solution

Without any additives, the color of CsSnI_3_ precursor solution was more yellowish than the solution with 10 mol% SnF_2_ additive, as presented in Fig. 6. This indicates that oxidation occurred more easily for pure CsSnI_3_.

**Fig. 7:**
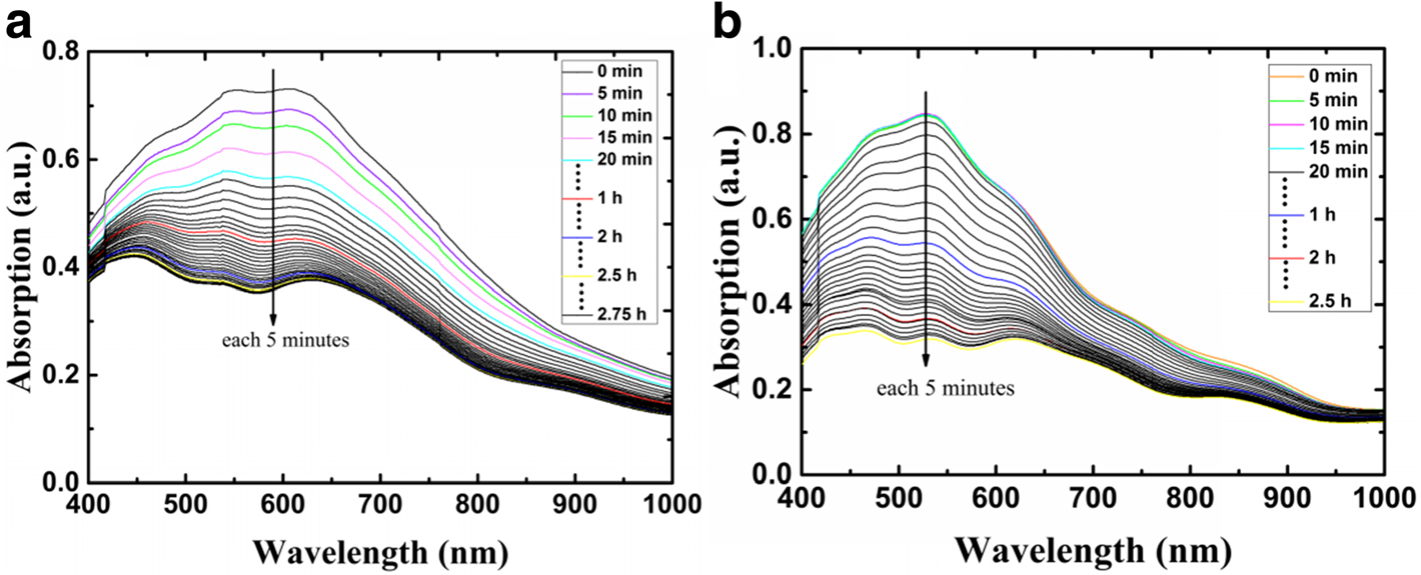
Absorption spectra of (**a**) pure CsSnI_3_ films and (**b**) CsSnI_3_ + 10% SnF_2_ films at different times in ambient air

We also plotted the evolution of absorption spectra at different time courses to investigate the degradation of CsSnI_3_ thin films with and without additives in air. As shown in Fig. 7, the black vertical line indicates the direction of change with increasing time in ambient air. Without additives, the CsSnI_3_ thin films degraded quickly when it was exposed to air at relative humidity of 57% and temperature of 13 °C. The degradation rate of the film was very fast in the beginning, but it slowed down a lot after 1 h. The degeneration process of CsSnI_3_ with 10 mol% SnF_2_ additive showed some difference. During the first few minutes, the film was quite stable and no oxidation occurred. Meanwhile the absorption peaks were at the same position. Few minutes later the oxidation rate accelerated and slowed down after an hour. Therefore, it can be deduced that the stability of the film is improved by the addition of SnF_2_.

## Conclusions

In summary, we have studied the different morphology characteristics of FASnI_3_ films prepared by using different anti-solvents and 10 mol% SnCl_2_. The aforementioned experimental results show that toluene and chlorobenzene are the best anti-solvents for improving the quality of the films and allow the film to completely cover the substrate. Using toluene as anti-solvent, we can gain the highest PCE of PSCs. The stability of FASnI_3_ can be kept for several hours, while CsSnI_3_ can only be stable for few minutes. So if we want to develop alternative Pb-free Sn-based perovskite films, the most critical issue is to stabilize the material, namely, suppressing the oxidation of Sn^2+^ within the crystal. This will enhance the long-term stable operation of perovskite films. This technique may offer a promising approach to fabricate the high efficiency of Pb-free Sn-based perovskite solar cell over the next few years.
